# Complex interactions between sperm viability and female fertility

**DOI:** 10.1038/s41598-019-51672-1

**Published:** 2019-10-25

**Authors:** Maximiliano Tourmente, C. Ruth Archer, David J. Hosken

**Affiliations:** 1 Centre for Ecology and Conservation, College of Life and Environmental Sciences, University of Exeter, Penryn, United Kingdom; 2 Institute of Evolutionary Ecology and Conservation Genomics, University of Ulm, Albert-Einstein-Allee 11, 89081 Ulm, Germany

**Keywords:** Sexual selection, Reproductive biology

## Abstract

Sperm viability is a major male fitness component, with higher sperm viability associated with enhanced sperm competitiveness. While many studies have focussed on sperm viability from the male fitness standpoint, its impact on female fitness is less clear. Here we used a panel of 32 isogenic *Drosophila simulans* lines to test for genetic variation in sperm viability (percentage of viable cells). We then tested whether sperm viability affected female fitness by mating females to males from low or high sperm viability genotypes. We found significant variation in sperm viability among genotypes, and consistent with this, sperm viability was highly repeatable within genotypes. Additionally, females mated to high sperm viability males laid more eggs in the first seven hours after mating, and produced more offspring in total. However, the early increase in oviposition did not result in more offspring in the 8 hours following mating, suggesting that mating with high sperm-viability genotypes leads to egg wastage for females shortly after copulation. Although mating with high sperm-viability males resulted in higher female fitness in the long term, high quality ejaculates would result in a short-term female fitness penalty, or at least lower realised fitness, potentially generating sexual conflict over optimal sperm viability.

## Introduction

Sperm competition, the competition between sperm from two or more males to fertilize a given set of ova^1^, is a powerful selective agent shaping the evolution of ejaculates^2^. Besides almost ubiquitous effects on testis size^3–7^ and sperm number^8–11^, adaptations to increase sperm competitiveness have been reported in a number of traits related to the quality of individual sperm themselves, such as morphology^3,12–17^, performance^18–22^, and metabolism^23,24^. For example, the proportion of viable sperm within an ejaculate (sperm viability) can affect ejaculate competitiveness and influence siring success. This is particularly well established in insects where sperm viability increases with sperm competition risk in comparative studies^25^, and predicts the outcome of competitive fertilizations intraspecifically^26,27^. Additionally, males strategically adjust the viability of sperm in an ejaculate to perceived risks of sperm competition^28,29^ and environmental insults like pesticide exposure can reduce sperm viability^30^. While the impacts of sperm viability on male fitness are reasonably well established, it is less clear how sperm viability affects female fitness.

Females need sufficient numbers of viable sperm for full fertility (avoidance of sperm limitation) and this is one possible reason for the evolution of polyandry^31–33^. For example, the number of eggs a female *Drosophila melanogaster* can produce over a lifetime and the number of sperm stored after a single mating are of similar magnitude^34,35^, although variation in fertilization efficiency could mean that more than one sperm is required per egg fertilized^36^. This suggests that, with limited mating opportunities, low sperm viability might result in unfulfilled reproductive potential for females. Interestingly, ejaculate adaptations that increase male fitness may result in decreased female fitness, thereby creating sexual conflict over ejaculate quality (reviewed by Stockley^37^ and Edward *et al*.^38^).

Sexual conflict is widespread because the sexes are often under contrasting patterns of selection, such that each sex has different optima for a shared trait (intralocus sexual conflict) or different preferred outcomes of an intersexual interaction (interlocus sexual conflict). For example, many traits that improve male fitness because they are beneficial in male-male competition are costly for females^1,39–43^. For example, male cockroaches (*Naupoeta cinerea*) engage in female guarding behaviour even after becoming sperm depleted, which reduces female fitness via enforced monogamy^44^. There can also be sexual conflict over seminal proteins and penis morphology, which can increase sperm competitiveness, but reduce female fitness^45–47^. For example, the *D. melanogaster* seminal protein sex-peptide increases male sperm competitiveness but reduces female longevity^48^. Additionally, increased numbers^49^, size^15^, and velocity^19,20,50^ of sperm in an ejaculate can increase sperm competitiveness, but also result in an increased risk of polyspermy and subsequent ova loss for females^51^. Males can also allocate fewer but more competitive sperm to ejaculates, in which case females might face sperm limitation^52^. Finally, ejaculate quality might trade off with non-ejaculate traits that enhance female fitness such as nuptial gifts or parental care^53^. So, there are multiple ways that variance in ejaculate quality – including sperm viability - could at least in principle impact female fitness.

Here we assessed potential effects of sperm viability on female fitness in *D. simulans*. Sperm viability is a key determinant of ejaculate quality and sperm competitiveness for insects in general^25–28^, and *Drosophila* in particular, where males produce highly viability ejaculates in response to increased sperm competition risk^29^. The present study was conducted using isofemale lines, which allowed us to replicate genotypes and assess sperm viability across them - thereby testing for genetic variation in sperm viability^54^, while also testing female fitness after receiving an ejaculate from genotypes with different sperm viability. Sexual selection has been extensively studied in *D. simulans*^55–58^. Sperm competitiveness is heritable and positively correlated with male attractiveness^59^, and while attractiveness is subject to inbreeding depression, sperm viability is not^60^. We used a panel of *D. simulans* genotypes to test for the presence of genetic variation in sperm viability, and to test how ejaculate quality (measured as sperm viability) affected female fitness by mating females to genotypes that had low- or high-viability sperm.

## Methods

### Animals and husbandry

In order to capture a significant portion of the genetic variation of a natural population of *D. simulans*, gravid females collected from Greece in 2010 were used to establish 32 isolines via full-sib mating. Isolines have been maintained by housing groups of males and females in 115 × 45 mm vials with non-overlapping generations (approximately 50 adults per vial in two replicates per isoline for > 100 generations). All flies in this study were maintained in incubators at 25^o^ C on a 12/12-hour light/dark cycle with *ad libitum Drosophila* Jazz-Mix food (Fisher Scientific). Light CO_2_ anaesthesia was used for fly collection, movement, and transference, and before fly dissections. No anaesthesia was used on flies within 24 hours of any mating assays as this affects mating behaviour^61^, instead, flies were individually aspirated into their housing vials.

### Sperm viability assay

Two groups of 10 females and 5 males per isoline (hereafter, genotype) were established in vials (95 × 25 mm) containing an excess of food to produce eggs for subsequent experiments. Flies were transferred into new vials every 24 hours for seven consecutive days (i.e. one vial per day per group). Eggs from these vials were incubated and virgin males were collected from these vials and housed individually (in 95 × 25 mm vials). Five males per isoline were collected on each of seven consecutive days (from a different vial each day). These males were held individually until they were five days old and then the sperm viability of one random male per day per isoline (=seven males per genotype) was assessed.

To assay sperm viability, males were anaesthetized using CO_2_ and their seminal vesicles (site of mature sperm storage) were separated from the male reproductive tract, placed in 10 µl Beadle solution (128.3 mM NaCl, 4.7 mM KCl, 2.3 mM CaCl_2_^62^) on a microscope slide, and pierced using a 0.1 × 12 mm insect pin. Sperm were forced out of the vesicles by gently pressing the vesicles with the flat side of the needle. Sperm viability was assessed after incubation in a moist chamber in the dark for 55 minutes using a LIVE/DEAD sperm viability kit (Molecular Probes L-7011), which contains two fluorescence probes that bind to DNA^60,63^. The first probe (SYBR-14) emits green fluorescence and is actively incorporated by living cells, and the second probe (propidium iodide, PI) emits red fluorescence and can only enter cells with damaged membranes (i.e. non-viable sperm). Briefly, an aqueous staining solution was prepared by diluting 2.4 µl of SYBR-14 stock solution (1 mM in DMSO), and 4.5 µl of PI stock solution (2.4 mM in water) in 93.1 µl of Beadle’s solution (working concentrations: SYBR-14 24 µM, PI 108 µM). Then, 5 µl of the staining solution were added to the drop containing the sperm (final concentrations SYBR-14 8 µM, PI 36 µM), and incubated for 5 minutes in the dark.

**Figure 1:**
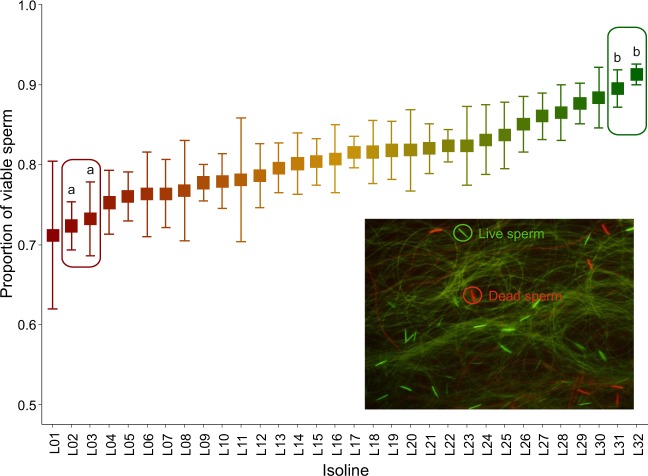
Proportion of viable sperm in 32 *D. simulans* genotypes (isolines). Squares represent means and whiskers standard errors. Square colors range from lower (red) to higher (green) mean sperm viability values. Different letters indicate significant differences (*p* < 0.05) between genotypes in a targeted *post-hoc* test (marginal means comparison). Lines selected for female fertility experiment are contained inside boxes, red lined box contains the “low sperm viability” genotypes and green lined box contains the “high sperm viability” genotypes. Insert: images of live and dead sperm cells after staining with LIVE/DEAD sperm viability probes.

Sperm were examined at ×400 magnification under phase contrast and fluorescence microscopy (BX61 Olympus), the latter employing a mercury excitation beam passed through 480/20 and 535/30 nm filters to assess green and red fluorescence respectively. Two images, one for each fluorescence filter, were captured from each field using a camera (DP70 Olympus) attached to the microscope, and subsequently merged (DP Manager v2.1.1.163 Olympus) to assess sperm status. A mean of 266 (±11.06 SE) sperm were counted (Cell Counter plugin ImageJ v.1.51j8) in at least 2 randomly chosen fields per sample and scored live (green) or dead (red) (see example in Fig. 1). Cells that were stained both green and red (less than 1% of sperm) were not counted. From our original sample of 224 males (1 male × 7 days × 32 isolines), 32 males were excluded because they generated samples with less than 40 visible cells (for a final sample size of 192 males). To test the reproducibility of our method a subsample of 19 males was recounted (10% of the original dataset) and a linear regression of the two sperm viability measures was conducted^64^. The association between the two measure was strong, with a slope close to unity and an intercept of close to zero, indicating high reproducibility (slope = 0.969, intercept = 0.004, *r*^2^ = 0.930, *F*_16,17 = _212.13, *p* < 0.0001).

### Female fitness: Assay 1

Once sperm viability for all genotypes (isolines) had been measured, we chose four extreme genotypes (two low sperm viability, two high sperm viability isolines) (Fig. 1), to test whether sperm viability had any effect on the fertility of females from a tester stock population (maintained in the lab with free mating at population size >1000 for >10 years). Thirty randomly chosen females from each of the high and low sperm viability isolines (housed in groups of 5/vial) were allowed to lay eggs for 24 hours. Virgin males (30 per isoline) were collected from these vials and housed individually for five days with excess food. Each male was then mated to a 4-day-old, virgin female collected from the outbred stock-population.

One day before mating, tester females were transferred to a vial (95 × 25 mm) containing 8 ml of Jazz mix food individually. The following day, males were introduced to females and pairs were left to interact for 7 hours. Then females were allowed to lay eggs for 8 days – this time period accurately captures *D. simulans* total fitness^55^. Since the mean remating interval for *D. simulans* females is approximately 2.7 days^36^, and remating frequency within 24 h following the initial mating is less than 2% in this species^65^, we assumed that the female reproductive output in the period of assessment corresponded to a single copulation with an experimental male. Initially females laid in egg counting vials (hereafter “initial period”) for seven hours (the time males were present), the number of eggs laid and the number of unhatched 20 h later were counted^66^, and this allowed the proportion of eggs hatching to be estimated. After this initial laying period, females were allowed to lay eggs in three consecutive sets of vials for 2, 3, and 3 days respectively. All sets of vials were monitored twice a day in the following 15 days for signs of larval activity and adult eclosion. After the first adult eclosion, each vial was monitored for 7 days and then frozen at −20 °C. The eclosed adults in each vial were counted and the total number of adult offspring per female was calculated as the sum of adults emerging from all four vials (hereafter “total offspring”). Eight females laid no eggs in the first seven hours and produced no offspring after 7 days of monitoring. These females were accounted as having failed to mate and were excluded from analysis, resulting in a final sample size of 112 females.

### Female fitness: Assay 2

In the previous experiment, we allowed females to copulate and lay eggs for 7 hours without monitoring copulation latency or duration. To test whether males from different sperm-viability group differed in the time taken to copulate or in copulation duration –which could potentially affect initial egg laying time in the previous experiment– we conducted a second series of investigations. Briefly, males were introduced to females and pairs were observed and copulation latency and duration were recorded (pairs not mating within 3 hours were discarded). Subsequently females were transferred to egg counting vials (vial 1) immediately after copulation ceased, where they were housed for 2 h, and then sequentially transferred to a new vial every 2 h (vials 2, 3 and 4), for a total of 4 vials per female and 8 h of egg-laying time (hereafter “initial period”). In addition, we extended the time assigned for hatching evaluation from 20 to 25 h (one hour more than the 100% eclosion time estimated in reference^66^) to ensure that the differences in egg hatching rates were not biased by different developmental times between sperm viability groups. The number of eggs laid in each vial and the number of unhatched 25 h later were counted, and the proportion of eggs hatching was estimated.

After the initial egg-laying period, females were allowed to lay eggs in three consecutive sets of vials (vials 5, 6, and 7) for 3, 3, and 2 days respectively. However, on the 5^th^ day post-mating, females were again transferred to two consecutive sets of egg laying vials (vials 8 and 9) for 2 h, for a total egg laying time of 4 h (hereafter “late assay period”), and then returned to the vial in which they were previously housed (vial 6). The number of eggs laid and the number of unhatched (25 h later) were counted in vials 8 and 9, and the proportion of hatched eggs was estimated. To test whether the egg-laying activity of the females at a later phase of the reproductive output differed from the initial period after mating, we compared the numbers of eggs laid and the hatching rate between the “late” assay period (vials 8, and 9) and a 4 h sub-interval of the initial period (vials 3 and 4, hereafter “early assay period”). We chose this combination of vials from the initial period since an extremely low number of females laid eggs in vials 1 and 2 (0 and 10 females respectively).

All sets of vials were monitored twice a day in the following 15 days for signs of larval activity and adult eclosion. After the first adult eclosion, each vial was monitored for 7 days and then frozen at −20 °C. The eclosed adults in each vial were counted and the total number of adult offspring per female was calculated as the sum of adults emerging from all eight vials (hereafter “total offspring”). A total of 117 females in two experimental blocks were assessed. Two females that died after mating and one female that mated but laid no eggs in the first seven hours and produced no offspring after 7 days of monitoring were excluded from analysis (final n = 114 females).

The total number of adult offspring that emerged in the 8 days post mating were combined from both fitness assays for statistical analyses. However, eggs laid in the first hours after mating were not combined across assays as we did not analyse egg number for Assay 2. This is because in Assay 2 we continually disturbed egg laying by moving females to new vials every 2 hours. Thus, the number of early eggs laid was not comparable to Assay 1, and not indicative of how many eggs an undisturbed female would lay. Nonetheless, the percentage of eggs hatched is still informative from Assay 2 data.

### Statistical analysis

All analyses were conducted using R version 3.6.0.^67^. The effect of genetic background on sperm viability was analysed with generalized mixed effect models (GLMM), including isoline as a fixed factor (32 levels), and day of analysis (7 levels) and egg-laying group (64 levels: two parental groups per isoline) as random factors. We also estimated the magnitude of genotypic effects upon the variability in sperm viability. Since our isolines have been kept in the lab for > 5 generations, calculating the broad-sense heritabilities (*h*^2^) would yield inaccurate estimates since inbreeding increases between line variance^68^. Instead, we calculated the repeatability of sperm viability as the intraclass coefficient (ICC)^69^, which is an estimator of the upper limit of heritability^70–72^. To estimate repeatability, we used a model similar to the one previously described for sperm viability, but included isoline as a random effect instead of as a fixed factor. However, since GLMMs do not provide a direct estimate of residual variance, and the interpretation of ICC estimations performed on GLMMs are only possible after transformation to the original data scale^64^, repeatability cannot be calculated for GLMMs using traditional approaches. Instead, we used the method described by Nakagawa *et al*.^73^ to estimate repeatability while accounting for the nonlinear relationships among the link scale and the scale of the data. The repeatability in the observation scale was estimated calculating the observation level variance as

$$1/(({{\rm{n}}}^{\ast }{{\rm{p}}}^{\ast }(1-{\rm{p}})))$$

where n = number of observations, and p = mean predicted viability value. This formula takes into account the specific logit link-function used in the model^73^. In addition, we compared sperm viability between four extreme isolines (two low sperm viability, two high sperm viability), by means of a targeted *post-hoc* test using the same GLMM model than for the original isoline panel, and a subsequent estimated marginal means test (emmeans package^74^).

The effect of sperm viability on mating success (second assay), latency to copulation (second assay), copulation duration (second assay), laid eggs (first assay and second assay separated), unhatched eggs (first assay), proportion of hatched eggs (first assay and second assay separated), offspring in the initial period (first assay), total offspring (first assay and second assay combined), and latency to copulation was analysed with GLMMs including sperm viability category (high or low) as a fixed factor, and isoline (4 levels) and egg-laying group nested within isoline (six parental groups per isoline) as random factors. When analysing data belonging to the second set of fertility experiments, we also included the experimental block (2 levels) as a random factor. When pooling data from the two fertility assays, we included the assay as a random factor and nested the blocks within. To test whether eggs laid and proportion of hatched eggs at the initial period after mating were similar to those on an equivalent time interval at a later phase of the egg-laying period (second assay only), we used a similar model than the ones detailed above with the addition of time and the interaction between time and sperm viability category as fixed effects. Time was included as a factor with two levels: early (vial 3 and 4) and late (vials 8 and 9).

Latency to copulation and copulation duration were analysed using Gamma distributions and “inverse” link functions (function “glmer” in the lme4 package^75^). The proportion of viable sperm, hatched eggs, and mating success were analysed using binomial distributions and “logit” link function. In the first two binomial models, the response variable was included in the model as a double vector containing the numbers of positive (i.e. sperm viability assay - viable sperm, fertility assays - hatched eggs) and negative (i.e. sperm viability assay - inviable sperm, fertility assays - unhatched eggs) results to account for the effect of the variable number of observations in the distribution of binomial data. Since these models showed over-dispersed data, we included an observation-level random effect nested within egg-laying group. Numbers of laid eggs, unhatched eggs, and offspring, were analysed using negative binomial distributions and “log” link functions (function “glmmTMB” in the glmmTMB package^76^). These variables were tested for overrepresentation of zero values (zero-inflation) by comparing the observed proportion of zeros in the data set with the proportion of zeros expected for simulated distributions with identical parameters. If zero-inflation was present, we applied a zero-inflation term to the corresponding models by including the sperm viability category as a fixed effect predicting zero-inflation. When data from the two fertility assays was pooled, we added the assay as a random predictor of zero-inflation. Significance of the fixed effects for the GLMMs was tested by comparing the fit of two different models that either include or exclude the fixed factor using a likelihood ratio test (LRT)^77^.

## Results

Sperm viability was variable across individual males with mean values ranging from 49–98% (Fig. 1). Male genotype significantly influenced sperm viability (GLMM: LRT *Χ*^2^ = 47.99 *p* = 0.026). This indicates significant genetic variation in sperm viability. Consistent with this, the repeatability of sperm viability, which sets an upper limit on the heritability^72^ of sperm viability, was high at 59%.

We chose four extreme male genotypes based on their mean sperm viability to test whether viability affected female fitness - two extremely low sperm viability genotypes (lines 02 and 03 – these had poor sperm viability and little within-genotype variation) - and two with very high sperm viability (lines 31 and 32) (Fig. 1). A targeted *post-hoc* test revealed significant differences in sperm viability between the two groups (GLMM: LRT *Χ*^2^ = 16.90, *p* = 0.0007), the “low viability group” averaging 73% (SE ± 2.9%) live sperm, and the “high viability” had a mean sperm viability of 90% (SE ± 1.4%).

**Figure 2:**
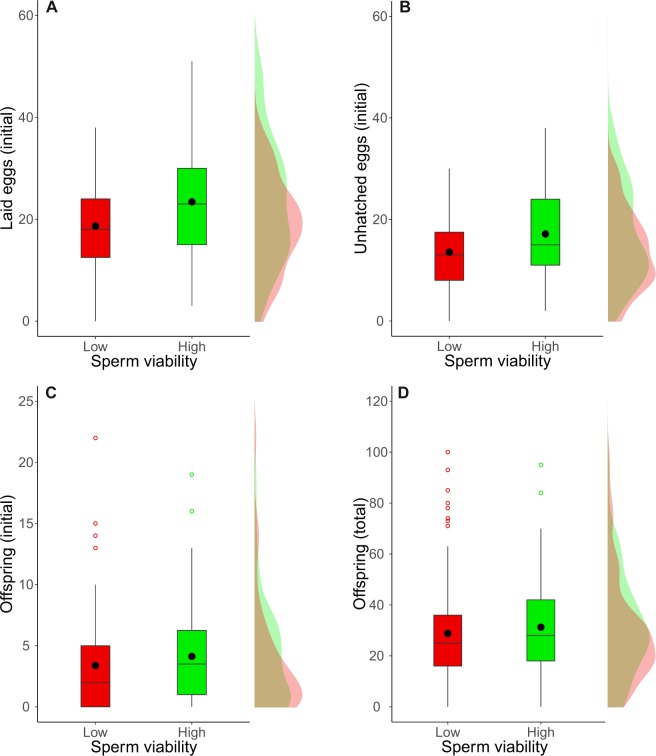
Sperm viability effect on *D. simulans* female fertility. (**A**) Numbers of eggs laid in the first seven hours post mating (first fertility assay). (**B**) Numbers of unhatched eggs from those laid in the first seven hours post mating (first fertility assay). (**C**) Numbers of adult offspring eclosing from the initial oviposition vial (first fertility assay). (**D**) Numbers of adult offspring eclosing from the total eight days of egg-laying (first + second fertility assays). Values for females mated to males from low and high sperm viability genotypes are represented in red and green colors respectively. Boxes correspond to the interquartile range (IQR), whiskers extend to the largest value no further than 1.5 * IQR from the limit of the box, black dots represent the mean, black bars represent the median, and empty dots represent outlier values (exceeding 3 standard deviations from the mean). Profiles at the right of each panel illustrate the distribution density of each sperm viability category.

In Assay 1 of female fitness, females mated with males from high sperm viability genotypes laid approximately 25% more eggs (18.67 ± 1.24 vs 23.42 ± 1.57 low and high sperm viability respectively) in the first seven hours than females mated with males from low sperm viability lines (Table 1, Fig. 2A). Sperm viability did not affect the numbers of females laying no eggs (non-significant zero-inflation factor, Table 1). Both groups had a similar percentage of eggs (~26%) hatching into larvae (Table 1). This occurs because, although females mated to high sperm viability males laid more eggs, they had significantly more unhatched eggs (13.56 ± 1.04 vs 17.14 ± 1.24, Table 1, Fig. 2B) (as well as weak non-significant trend of more hatched eggs: 5.11 ± 0.75 vs 6.28 ± 0.69, GLMM: LRT *Χ*^2^ = 1.58, *p* = 0.21). Furthermore, the numbers of adult offspring emerging from eggs laid in the first seven hours after mating (vial 1) were similar between the two sperm viability groups (Table 1, Fig. 2C). This tentatively suggests more death occurred during the larval or pupal stages in the high sperm-viability treatment: they laid more eggs, but had the same number of adult offspring emerge during the first 7 hours after mating as the low sperm viability group.

**Table 1:** Sperm viability effects on *D. simulans* female fertility.

Dependent variable	Low viability	High viability	Independent variable	*z*-value	Variance	*X* ^2^	*p*
Laid eggs	18.67 ± 1.24	23.42 ± 1.57	Viability category (Fixed)	−2.190		3.95	0.047
(Initial, first assay)			Genotype (Random)		<0.001		
			Egg-laying group (Random)		<0.001		
Unhatched eggs	13.56 ± 1.04	17.14 ± 1.24	Viability category (Fixed)	−2.160		4.33	0.037
(Initial, first assay)			Genotype (Random)		<0.001		
			Egg-laying group (Random)		<0.001		
Proportion hatched eggs	0.26 ± 0.03	0.26 ± 0.02	Viability category (Fixed)	−0.252		0.06	0.802
(Initial, first assay)			Genotype (Random)		0.018		
			Egg-laying group (Random)		0.114		
			Observation (Random)		0.859		
First vial offspring	3.38 ± 0.61	4.13 ± 0.56	Viability category (Fixed)	−0.443		0.19	0.660
(Initial, first assay)			Genotype (Random)		0.073		
			Egg-laying group (Random)		<0.001		
			Viability category (ZI - Fixed)^a^	0.254		0.06	0.801
Mating success (%)	57.89	44.35	Viability category (Fixed)	2.061		3.26	0.071
(Second assay)			Genotype (Random)		<0.001		
			Egg-laying group (Random)		<0.001		
			Block (Random)		0.052		
Latency to copulation (min)	75.24 ± 6.36	75.54 ± 6.51	Viability category (Fixed)	0.074		0.01	0.941
(Second assay)			Genotype (Random)		<0.001		
			Egg-laying group (Random)		<0.001		
			Block (Random)		<0.001		
Copulation duration (min)	20.27 ± 0.60	21.30 ± 0.91	Viability category (Fixed)	−2.800		0.08	0.780
(Second assay)			Genotype (Random)		<0.001		
			Egg-laying group (Random)		<0.001		
			Block (Random)		<0.001		
Proportion hatched eggs	0.42 ± 0.06	0.48 ± 0.06	Viability category (Fixed)	−1.390		1.98	0.160
(Initial, second assay)			Genotype (Random)		<0.001		
			Egg-laying group (Random)		<0.001		
			Observation (Random)		0.423		
			Block (Random)		<0.001		
Total offspring	28.79 ± 1.88	31.26 ± 1.93	Viability category (Fixed)	−2.234		4.12	0.042
(First + second assays)			Genotype (Random)		<0.001		
			Egg-laying group (Random)		0.015		
			Block (Random)		0.020		
			Assay (Random)		0.008		
			Viability category (ZI - Fixed)^a^	−1.312		2.02	0.155
			Assay (ZI - Random)		5.46		

Values shown here (mean ± standard error) are the outcomes for singly mating females with males from low and high sperm viability genotypes. Statistical parameters for numbers of laid eggs (Wald’s *z*-value and variance), unhatched eggs, and offspring were estimated using a GLMM with negative binomial distribution and “log” link function, while in the case of proportion of hatching eggs a GLMM with binomial distribution and “logit” link function was used. *X*^2^ and *p* valuesfor fixed factors correspond to likelihood ratio tests between the full model and a restricted model excluding the fixed factor. ^a^ZI: zero inflation term for datasets with higher numbers of zero values than predicted by the specified distribution.

In Assay 2 of female fitness, there were no significant differences in mating success, copulation latency or copulation duration between high or low sperm viability males (Table 1). Thus, differences described above are unlikely to be associated to differences in the timing of mating events. Additionally, these assays revealed that although the proportion of hatched eggs (0.45 ± 0.06) was higher when assessed 25 h after oviposition (Assay 2) than when assessed after 20 h (Assay 1), again the proportion of hatched eggs did not differ between sperm viability groups (Table 1). This suggests that although assessing egg hatching at 20 h post-laying might underestimate the total proportion of hatched eggs; it does not bias the estimation with respect to sperm viability. This is true shortly after mating (4–8 h after mating) and 5 days after mating and oviposition (Table 2).

**Table 2:** Effects of sperm viability and time of egg laying after mating on *D. simulans* female fertility.

Dependent variable	Low viability		High viability		Independent variable	*z*	Variance	*X* ^2^	*p*
	Early	Late	Early	Late		value			
Laid eggs	2.83 ± 0.51	3.22 ± 0.47	2.43 ± 0.28	3.10 ± 0.42	Viability category (Fixed)	1.438		1.00	0.317
(Second assay)					Time of egg laying (Fixed)	0.994		0.26	0.608
					Viability * Time (Fixed)	−0.923		0.90	0.344
					Genotype (Random)		<0.001		
					Egg-laying group (Random)		<0.001		
					Female (Random)		0.006		
					Block (random)		0.097		
					Viability category (ZI - Fixed)^a^	1.106		2.01	0.126
Prop. hatched eggs	0.42 ± 0.06	0.38 ± 0.05	0.49 ± 0.04	0.43 ± 0.06	Viability category (Fixed)	1.764		3.16	0.076
(Second assay)					Time of egg laying (Fixed)	1.396		1.91	0.167
					Viability * Time (Fixed)	0.822		0.68	0.410
					Genotype (Random)		<0.001		
					Egg-laying group (Random)		0.113		
					Female (Random)		0.187		
					Block (random)		0.038		

Values shown here (mean ± standard error) correspond to eggs laid in two 4 h intervals (early: 4 h after mating, late: 5 days after mating) after singly mating virgin females with males from low and high sperm viability genotypes in the second set of mating assays. Statistical parameters (Wald’s *z*-value and variance) for numbers of eggs laid and offspring were estimated using a GLMM with negative binomial distribution and “log” link function, while in the case of proportion of hatching eggs a GLMM with binomial distribution and “logit” link function was used. *X*^2^ and *p* valuesfor fixed factors correspond to likelihood ratio tests between the full model and a restricted model excluding the fixed factor. ^a^ZI: zero inflation term for datasets with higher numbers of zero values than predicted by the specified distribution.

Combining data from the two fertility assays to compare female fitness, revealed females mated with high sperm viability genotypes showed a small but significant increase in the numbers of offspring produced over the complete 7-day laying period (low sperm viability: 28.79 ± 1.88, high sperm viability: 31.26 ± 1.93, Table 1, Fig. 2D) and sperm viability did not affect the number of females that produced zero adult offspring (non-significant zero-inflation factor, Table 1).

## Discussion

Sperm viability varied significantly among *D. simulans* genotypes (isolines), and its repeatability was consistent with the heritability of sperm competitiveness previously reported in this taxon^59^. Similar results have been reported across other studies too^78–81^. Perhaps more interestingly, when females were mated with males from high sperm viability genotypes they laid more eggs in the first seven hours after mating, and produced more offspring in total. However, the short-term increase in oviposition (7 hours of laying) did not result in more offspring initially, suggesting females mated to males with high sperm viability wasted eggs shortly after copulation. This is despite the fact that mating with high sperm-viability males ultimately resulted in higher female fitness.

The short-term finding suggests that high sperm viability elevates the initial egg laying response in females. Although virgin *Drosophila* females are able to ovulate and lay (infertile) eggs at a very low rate, mating drastically and rapidly increases ovulation^82^. Both sperm and seminal-fluid proteins (Sfps) contribute to this effect, which is not as pronounced if one of these components is not present^35,83–85^. Although few studies have investigated how sperm (independently of Sfps) in the female reproductive tract affect ovulation, a possible mechanism explaining our finding has been described in butterflies where sperm activate mechano-receptors in the female tract to stimulate egg laying^86^. Alternatively, more viable sperm could result in more Sfps being transferred to females because Sfps can be attached to *Drosophila* sperm^87^. For example, in the closely related *D. melanogaster*, sex-peptide (one key Sfp) is bound to sperm and cleaved and released upon female sperm storage, triggering and sustaining ovulatory responses^88,89^, and *D. simulans* has a sex-peptide analog^90^. Either mechanism (binding or mechano-reception) could generate our results since it appears sperm have to actively enter the female sperm storage organs (sperm receptacle and spermatheca: collectively SSOs)^36^ and thus more viable sperm would result in more sperm storage, and consequently a higher degree of mechanical stimulation and/or higher concentrations of Sfps in the system. Although either of these outcomes could in turn promote a more exaggerated ovulatory response, the exact mechanism involved was not the focus of this study. However, it must be noted that *D. simulans* females only store about 30% of the sperm in an ejaculate and eject the rest before oviposition commences^36^. Thus, inviable sperm may never reach the SSOs and the sperm population ultimately stored may all be of high viability even if they come from ejaculates with different proportions of viable sperm. This would suggest a pre-storage overstimulation mechanism (mechanical or otherwise), in which the totality of the ejaculate might affect the female physiological state, as the more plausible alternative.

Nonetheless, in the short term more viable sperm resulted in more eggs laid, but no increase in offspring. This is possibly because, in many *Drosophila* species, females initiate ovulation and egg release before completing sperm storage^35,82^. These early-ovulated eggs are less likely to develop than eggs laid at later points^90,91^, suggesting that they may reach the fertilization site before sperm are ready to fertilize^35^. Thus a general acceleration of ovulation can decrease the efficiency of fertilization, explaining the increased number of unhatched eggs shortly after matings with high sperm-viability males. This suggests mating to high sperm-viability genotypes results in a short-term resource wastage for females. However, females mated to high sperm viability males also produced more offspring across the whole assay period: they had higher fitnesss. This is consistent with sperm viability determining the fertilizing capacity of a sperm population^92,93^, and decreased female fertility when viable sperm are depleted from SSOs^91,94,95^.

Associations among different sperm traits are generally not clear, with studies finding strongly positive^79,96^, to negative^81,97^, and no^98,99^ correlations between characters thought to determine sperm quality. Moreover, the recruitment, maintenance, and usage of the sperm population in the SSOs of female *Drosophila* mainly depend on the female nervous system, SSO secretions, and male secreted SfPs^91^. This suggests that associations between the viability of ejaculated sperm and the long-term viability of the sperm in the SSOs can be complex. Suprisingly, our results appear to indicate a clear link, and that an ejaculate containing more viable sperm results in the establishment of a more fertile sperm reserve in the longer term.

While high sperm viability ultimately resulted in higher female fitness, the initial egg wastage at least represents “potential fitness” lost, and egg loss cannot be cheap. In fact one reason for the general lack of female secondary sex-traits is the cost of egg production^100–102^. It therefore appears that a trait likely to be beneficial in sperm competition (sperm viability) has short-term costs for females, and thus there is likely to be some sexual conflict over sperm viability^38^. This is consistent with conflict being characterised as males and females agreeing on the destination, but not on how to get there^103^, and some male-female conflict over optimal sperm viability is consistent with the lack of inbreeding depression for it^60^. Inbreeding depression requires directional dominance, which results from a history of directional selection on a trait^71,72,104^, and lack of inbreeding depression can be a signal of stabilising selection. Since sexual conflict can act as a form of balancing selection^105^, our results might be indicative of net-stabilising selection on sperm viability – good for males but bad for females – which offers a tentative explanation for the lack of inbreeding depression in viability previously documented^60,106^. As noted however, female fitness was ultimately greater with high sperm viability despite the initial egg loss. How this would play out with multiple mating is not clear.

Overall, our study shows significant genetic variation and highly repeatable sperm quality in *D. simulans*, and that ejaculates with highly competitive characteristics result in short term egg wastage. However, securing ejaculates with high sperm viability ultimately resulted in more offspring and higher female fitness. How males with higher sperm viability are able to cause females to waste resource and have higher fitness is not clear, but logic suggests there must be a female cost somewhere – it does not seem feasible to us that less efficiency generates higher output in a biological system, unless this is the optimal female response to the male “game” for example^107^. This requires further investigation.

## Data Availability

The datasets generated and analysed during the current study have been uploaded on Dryad and are available for download: 10.5061/dryad.n8pk0p2qz.

## References

[1] Parker GA (1970). Sperm competition and its evolutionary consequences in insects. Biol Rev.

[2] Birkhead, T. R., Hosken, D. J. & Pitnick, S. *Sperm**Biology: An Evolutionary Perspective*. (Academic Press, 2009).

[3] Gage MJG (1994). Associations between body size, mating pattern, testis size and sperm lengths across butterflies. Proc R Soc B.

[4] Stockley P, Gage MJ, Parker GA, Møller AP (1997). Sperm competition in fishes: The evolution of testis size and ejaculate characteristics. Am Nat.

[5] Hosken DJ (1997). Sperm competition in bats. Proc R Soc B.

[6] Hosken DJ, Ward PI (2001). Experimental evidence for testis size evolution via sperm competition. Ecol Lett.

[7] Firman RC, Simmons LW (2008). The frequency of multiple paternity predicts variation in testes size among island populations of house mice. J Evol Biol.

[8] Immler S (2011). Resolving variation in the reproductive tradeoff between sperm size and number. Proc Natl Acad Sci USA.

[9] Tourmente M, Delbarco Trillo J, Roldan ERS (2015). No evidence of trade-offs in the evolution of sperm numbers and sperm size in mammals. J Evol Biol.

[10] Aron S (2016). Sperm production characteristics vary with level of sperm competition in *Cataglyphis* desert ants. Funct Ecol.

[11] Liao WB (2018). Ejaculate evolution in external fertilizers: Influenced by sperm competition or sperm limitation?. Evolution.

[12] Johnson DDP, Briskie JV (1999). Sperm competition and sperm length in shorebirds. Condor.

[13] Byrne PG, Simmons LW, Roberts JD (2003). Sperm competition and the evolution of gamete morphology in frogs. Proc R Soc Lond B Biol Sci.

[14] Balshine S, Leach BJ, Neat F, Werner NY, Montgomerie R (2001). Sperm size of African cichlids in relation to sperm competition. Behav Ecol.

[15] Gage MJG, Morrow EH (2003). Experimental evidence for the evolution of numerous, tiny sperm via sperm competition. Curr Biol.

[16] Tourmente M, Gomendio M, Roldan ERS, Giojalas LC, Chiaraviglio M (2009). Sperm competition and reproductive mode influence sperm dimensions and structure among snakes. Evolution.

[17] Tourmente M, Gomendio M, Roldan ERS (2011). Sperm competition and the evolution of sperm design in mammals. BMC Evol Biol.

[18] Birkhead TR, G. MJ, Burke T, Froman DP (1999). Sperm mobility determines the outcome of sperm competition in the domestic fowl. Proc R Soc Lond B Biol Sci.

[19] Gage MJG (2004). Spermatozoal traits and sperm competition in Atlantic salmon: relative sperm velocity is the primary determinant of fertilization success. Curr Biol.

[20] Fitzpatrick JL (2009). Female promiscuity promotes the evolution of faster sperm in cichlid fishes. Proc Natl Acad Sci USA.

[21] Gasparini C, Simmons LW, Beveridge M, Evans JP (2010). Sperm swimming velocity predicts competitive fertilization success in the green swordtail *Xiphophorus helleri*. PLoS One.

[22] Tourmente M, Varea-Sanchez M, Roldan ERS (2019). Faster and more efficient swimming: energy consumption of murine spermatozoa under sperm competition. Biol Reprod.

[23] Tourmente M, Villar-Moya P, Rial E, Roldan ERS (2015). Differences in ATP generation via glycolysis and oxidative phosphorylation and relationships with sperm motility in mouse species. J Biol Chem.

[24] Tourmente M (2015). Performance of rodent spermatozoa over time is enhanced by increased ATP concentrations: the role of sperm competition. Biol Reprod.

[25] Hunter FM, Birkhead TR (2002). Sperm viability and sperm competition in insects. Curr Biol.

[26] Fry CL, Wilkinson GS (2004). Sperm survival in female stalk-eyed flies depends on seminal fluid and meiotic drive. Evolution.

[27] Garcia-Gonzalez F, Simmons LW (2005). Sperm viability matters in insect sperm competition. Curr Biol.

[28] Thomas ML, Simmons LW (2007). Male crickets adjust the viability of their sperm in response to female mating status. Am Nat.

[29] Moatt JP, Dytham C, Thom MD (2014). Sperm production responds to perceived sperm competition risk in male *Drosophila melanogaster*. Physiol Behav.

[30] Chaimanee V, Evans JD, Chen Y, Jackson C, Pettis JS (2016). Sperm viability and gene expression in honey bee queens (*Apis mellifera*) following exposure to the neonicotinoid insecticide imidacloprid and the organophosphate acaricide coumaphos. J Insect Physiol.

[31] Arnqvist G, Nilsson T (2000). The evolution of polyandry: multiple mating and female fitness in insects. Anim Behav.

[32] Price TAR, Hodgson DJ, Lewis Z, Hurst GDD, Wedell N (2008). Selfish genetic elements promote polyandry in a fly. Science.

[33] Alonzo SH, Pizzari T (2013). Selection on female remating interval is influenced by male sperm competition strategies and ejaculate characteristics. Philos Trans R Soc Lond B Biol Sci.

[34] Lefevre G, Jonsson UB (1962). Sperm transfer, storage, displacement, and utilization in *Drosophila melanogaster*. Genetics.

[35] Bloch Qazi MC, Heifetz Y, Wolfner MF (2003). The developments between gametogenesis and fertilization: ovulation and female sperm storage in *Drosophila melanogaster*. Dev Biol.

[36] Manier MK (2013). Rapid diversification of sperm precedence traits and processes anmong three sibling *Drosophila* species. Evolution.

[37] Stockley P (1997). Sexual conflict resulting from adaptations to sperm competition. Trends Ecol Evol.

[38] Edward DA, Stockley P, Hosken DJ (2014). Sexual conflict and sperm competition. Cold Spring Harbor perspectives in biology.

[39] Rice WR (1996). Sexually antagonistic male adaptation triggered by experimental arrest of female evolution. Nature.

[40] Alonzo SH, Warner RR (1999). A trade-off generated by sexual conflict: Mediterranean wrasse males refuse present mates to increase future success. Behav Ecol.

[41] Martin OY, Hosken DJ (2003). The evolution of reproductive isolation through sexual conflict. Nature.

[42] Arnqvist, G. & Rowe, L. *Sexual conflict*. (Princeton University Press, 2005).

[43] Okada K, Katsuki M, Sharma MD, House CM, Hosken DJ (2014). Sexual conflict over mating in *Gnatocerus cornutus*? Females prefer lovers not fighters. Proc R Soc B.

[44] Montrose VT, Harris WE, Moore PJ (2003). Sexual conflict and cooperation under naturally occurring male enforced monogamy. J Evol Biol.

[45] Chapman T, Liddle LF, Kalb JM, Wolfner MF, Partridge L (1995). Cost of mating in *Drosophila melanogaster* females is mediated by male accessory gland products. Nature.

[46] Civetta A, Clark AG (2000). Correlated effects of sperm competition and postmating female mortality. Proc Natl Acad Sci USA.

[47] Hotzy C, Arnqvist G (2009). Sperm competition favors harmful males in seed beetles. Curr Biol.

[48] Wigby S, Chapman T (2005). Sex peptide causes mating costs in female *Drosophila melanogaster*. Curr Biol.

[49] Wedell N, Cook PA (1999). Butterflies tailor their ejaculate in response to sperm competition risk and intensity. Proc R Soc B.

[50] Gomez Montoto L (2011). Sperm competition differentially affects swimming velocity and size of spermatozoa from closely related muroid rodents: head first. Reproduction.

[51] Snook RR, Hosken DJ, Karr TL (2011). The biology and evolution of polyspermy: insights from cellular and functional studies of sperm and centrosomal behavior in the fertilized egg. Reproduction.

[52] Wedell N, Gage MJG, Parker GA (2002). Sperm competition,male prudence and sperm-limited females. Trends Ecol Evol.

[53] Simmons LW, Craig M, Llorens T, Schinzig M, Hosken D (1993). Bushcricket spermatophores vary in accord with sperm competition and parental investment theory. Proc R Soc B.

[54] Lupold S (2012). How multivariate ejaculate traits determine competitive fertilization success in *Drosophila melanogaster*. Curr Biol.

[55] Taylor ML, Wedell N, Hosken DJ (2007). Sexual selection and female fitness in *Drosophila simulans*. Behav Ecol Sociobiol.

[56] Sharma MD, Tregenza T, Hosken DJ (2010). Female mate preferences in *Drosophila simulans*: evolution and costs. J Evol Biol.

[57] Maraqa MS (2017). Constrained evolution of the sex comb in *Drosophila simulans*. J Evol Biol.

[58] Archer CR, Stephens RM, Sharma MD, Hosken DJ (2017). The *Drosophila simulans* Y chromosome interacts with the autosomes to influence male fitness. J Evol Biol.

[59] Hosken DJ, Taylor ML, Hoyle K, Higgins S, Wedell N (2008). Attractive males have greater success in sperm competition. Curr Biol.

[60] Okada K, Blount JD, Sharma MD, Snook RR, Hosken DJ (2011). Male attractiveness, fertility and susceptibility to oxidative stress are influenced by inbreeding in *Drosophila simulans*. J Evol Biol.

[61] Greenspan, R. J. *Fly Pushing: The Theory and Practice of Drosophila Genetics*. (Cold Spring Harbor Laboratory Press, 1997).

[62] Bressac C, Chevrier C (1998). Offspring and sex ratio are independent of sperm management in *Eupelmus orientalis* females. J Insect Physiol.

[63] Holman L (2009). *Drosophila melanogaster* seminal fluid can protect the sperm of other males. Funct Ecol.

[64] Nakagawa S, Schielzeth H (2010). Repeatability for Gaussian and non-Gaussian data: a practical guide for biologists. Biol Rev Camb Philos Soc.

[65] Markow TA (2002). Perspective: female remating, operational sex ratio, and the arena of sexual selection in *Drosophila* species. Evolution.

[66] Markow TA, Beall S, Matzkin LM (2009). Egg size, embryonic development time and ovoviviparity in *Drosophila* species. J Evol Biol.

[67] R. A Language and Environment for Statistical Computing v. 3.4.0 (R Foundation for Statistical Computing, Vienna, Austria, 2017).

[68] David JR (2005). Isofemale lines in *Drosophila*: an empirical approach to quantitative trait analysis in natural populations. Heredity.

[69] Hoffmann AA, Parsons PA (1988). The analysis of quantitative variation in natural populations with isofemale strains. Genet Sel Evol.

[70] Boake CRB (1989). Repeatability: its role in evolutionary studies of mating behavior. Evol Ecol.

[71] Falconer, D. S. & Mackay, T. F. *Introduction to Quantitative Genetics*. 4th edn, (Longmans Green, 1996).

[72] Lynch, M. & Walsh, B. *Genetics and Analysis of Quantitative Traits*. (Oxford University Press, 1998).

[73] Nakagawa S, Johnson PCD, Schielzeth H (2017). The coefficient of determination R(2) and intra-class correlation coefficient from generalized linear mixed-effects models revisited and expanded. Journal of the Royal Society, Interface.

[74] Estimated Marginal Means, aka Least-Squares Means v. 1.3.2 (2019).

[75] Bates, D., Mächler, M., Bolker, B. & Walker, S. Fitting linear mixed-effects models using lme4. *J Stat Soft* **67**, 10.18637/jss.v067.i01 (2015).

[76] Brooks ME (2017). glmmTMB balances speed and flexibility among packages for zero-inflated generalized linear mixed modeling. R J.

[77] Luke SG (2017). Evaluating significance in linear mixed-effects models in R. Behav Res Methods.

[78] Morrow EH, Gage MJG (2001). Artificial selection and heritability of sperm length in *Gryllus bimaculatus*. Heredity.

[79] Moore PJ, Harris WE, Montrose VT, Levin D, Moore AJ (2004). Constraints on evolution and postcopulatory sexual selection: trade-offs among ejaculate characteristics. Evolution.

[80] Harris WE, Moore AJ, Moore PJ (2007). Variation in sperm size within and between ejaculates in a cockroach. Funct Ecol.

[81] Birkhead TR, Pellat EJ, Brekke P, Yeates R, Castillo-Juarez H (2005). Genetic effects on sperm design in the zebra finch. Nature.

[82] Heifetz Y, Lung O, Frongillo EA, Wolfner MF (2000). The *Drosophila* seminal fluid protein Acp26Aa stimulates release of oocytes by the ovary. Curr Biol.

[83] Avila FW, Bloch Qazi MC, Rubinstein CD, Wolfner MF (2012). A requirement for the neuromodulators octopamine and tyramine in *Drosophila melanogaster* female sperm storage. Proc Natl Acad Sci USA.

[84] Rubinstein CD, Wolfner MF (2013). *Drosophila* seminal protein ovulin mediates ovulation through female octopamine neuronal signaling. Proc Natl Acad Sci USA.

[85] Mattei AL, Riccio ML, Avila FW, Wolfner MF (2017). Integrated 3D view of postmating responses by the *Drosophila melanogaster* female reproductive tract, obtained by micro-computed tomography scanning. Proc Natl Acad Sci USA.

[86] Obara Y (1975). Mating behavior of the cabbage whithe butterfly, *Pieris rapae crucivora*. J Comp Physiol.

[87] Kubli, E. In *Advances in Developmental Biochemistry* Vol. 4 (ed Paul M. Wassarman) 99–128 (Academic Press, 1996).

[88] Liu H, Kubli E (2003). Sex-peptide is the molecular basis of the sperm effect in *Drosophila melanogaster*. Proc Natl Acad Sci USA.

[89] Singh A (2018). Long-term interaction between *Drosophila* sperm and sex peptide is mediated by other seminal proteins that bind only transiently to sperm. Insect Biochem Mol Biol.

[90] Chapman T, Herndon LA, Heifetz Y, Partridge L, Wolfner MF (2001). The Acp26Aa seminal fluid protein is a modulator of early egg hatchability in *Drosophila melanogaster*. Proc R Soc B.

[91] Schnakenberg SL, Siegal ML, Bloch Qazi MC (2012). Oh, the places they’ll go: Female sperm storage and sperm precedence in Drosophila melanogaster. Spermatogenesis.

[92] Damiens D, Bressac C, Brillard JP, Chevrier C (2002). Qualitative aspects of sperm stock in males and females from *Euplemus orientalis* and *Dinarmus basalis* (Hymenoptera: Chalcidoidea) as revealed by dual fluorescence. Physiol Entomol.

[93] Collins AM, Donoghue AM (1999). Viability assessment of honey bee, *Apis mellifera*, sperm using dual fluorescent staining. Theriogenology.

[94] Collins AM (2004). Functional longevity of honey bee, *Apis mellifera*, queens inseminated with low viability semen. J Apic Res.

[95] Reinhardt K, Ribou A-C (2013). Females become infertile as the stored sperm’s oxygen radicals increase. Scientific reports.

[96] Gomez Montoto L (2011). Sperm competition, sperm numbers and sperm quality in muroid rodents. PLoS One.

[97] Taborsky M, Schütz D, Goffinet O, Sander van Doorn G (2018). Alternative male morphs solve sperm performance/longevity trade-off in opposite directions. Science Advances.

[98] Simmons LW, Kotiaho JS (2002). Evolution of ejaculates: patterns of phenotypic and genotypic variation and condition dependence in sperm competition traits. Evolution.

[99] Damiens D, Bressac C, Chevrier C (2003). The effect of age on sperm stock and egg laying in the parasitoid wasp, *Dinarmus basalis*. J Insect Sci.

[100] Wang Y, Salmon AB, Harshman LG (2001). A cost of reproduction: oxidative stress succeptibility is associated with increased egg production in *Drosophila melanogaster*. Experimental gerontology.

[101] Yang CH, Belawat P, Hafen E, Jan LY, Jan YN (2008). *Drosophila* egg-laying site selection as a system to study simple decision-making processes. Science.

[102] Hosken DJ, Alonzo SH, Wedell N (2016). Why aren’t signals of female quality more common?. Anim Behav.

[103] Hosken DJ, Archer CR, Mank JE (2019). Sexual conflict. Curr Biol.

[104] Roff, D. A. *Evolutionary Quantitative Genetics*. (Chapman and Hall, 1997).

[105] Mank JE (2017). Population genetics of sexual conflict in the genomic era. Nature Rev Genet.

[106] Ala-Honkola O (2013). Inbreeding reveals mode of past selection on male reproductive characters in *Drosophila melanogaster*. Ecology and evolution.

[107] McNamara JM, Houston AI (2002). Credible threats and promises. Philos Trans R Soc Lond B Biol Sci.

